# Advances and mechanisms in the application and treatment of functionalized stem cells and their derivatives in allergic rhinitis

**DOI:** 10.3389/fped.2026.1931883

**Published:** 2026-09-28

**Authors:** Lijun Yu, Xiaojuan Xu, Haihua Chen, Bingbing Li, Lian Feng

**Affiliations:** Department of Pediatrics, Shaoxing Central Hospital, Affiliated Central Hospital of Shaoxing University, Shaoxing, Zhejiang, China

**Keywords:** allergic rhinitis, exosomes, functionalization, immune regulation, mesenchymal stem cells

## Abstract

Allergic rhinitis (AR) is characterized by Th2/Th17/Treg immune imbalance and nasal mucosal barrier damage, and current treatments struggle to restore immune homeostasis. Mesenchymal stem cells (MSCs) and their exosomes can exert anti-inflammatory and barrier-repairing effects through paracrine pathways, but natural MSCs have poor targeting and short local retention, limiting their efficacy. Recent functionalization and novel delivery systems have significantly enhanced their efficacy, and combination therapy strategies have shown synergistic potential. However, current evidence is largely derived from animal models, clinical studies lack large-scale, long-term data, and standardization of MSCs from different sources and engineered products is insufficient. Future research should leverage multi-omics and intelligent design to advance personalized treatment plans, providing a new direction for non-hormonal “immune reset” therapy for AR. This review systematically summarizes the mechanisms, strategies, and translational bottlenecks of functionalized stem cell derivatives in AR, providing a theoretical framework for subsequent precision cell therapy research.

## 1 Introduction

Allergic rhinitis (AR) is one of the most common chronic diseases worldwide, especially among children and adolescents, and its incidence is currently on the rise (1, 2). Allergic rhinitis is essentially an inflammatory condition of the nasal mucosa triggered by inhaled allergens, often manifesting as nasal itching, frequent sneezing, increased secretions, and nasal congestion (3). In addition to nasal symptoms, ocular symptoms are also common, such as itchy or red eyes and tearing. These patients are also prone to respiratory symptoms such as coughing and wheezing attacks, severely impacting their quality of life (4–6).

The current mainstream treatment for allergic rhinitis focuses on environmental control, drug therapy, and allergen-specific immunotherapy (AIT), with drug therapy being the most common in clinical practice (7, 8). However, the long-term efficacy and application of existing allergic rhinitis treatment strategies are limited by factors such as differences in recommendations among different guidelines, insufficient evidence of efficacy for some drugs, poor patient compliance and long-term standardized medication, large differences in clinical phenotypes leading to insufficient individualized treatment, and the accessibility and economic burden of immunotherapy and biologics (9–11). Stem cells are multipotent stromal cells derived from the mesoderm. They have the potential to differentiate into a variety of cell lineages. They are widely distributed in a variety of organs and tissues and have both immunomodulatory functions and tissue repair capabilities (12, 13). Treatment strategies for allergic rhinitis mostly focus on symptom control, while intervention in the immune imbalance mechanism of the disease is relatively limited. Compared with existing solutions, the advantage of stem cell therapy is that it may intervene in the pathological process from an immune regulatory level, rather than just being limited to symptomatic relief (14). If we can promote the reconstruction of immune homeostasis through mechanism regulation, it will not only help to improve acute phase performance, but may also reduce the risk of long-term recurrence. Therefore, it has become one of the research directions with exploratory value in recent years.

Although numerous studies have confirmed the anti-inflammatory and immunomodulatory effects of mesenchymal stem cells (MSCs) and their derivatives in AR animal models, several key gaps remain in the existing evidence (15–17). The immunomodulatory function of MSCs is bidirectional and microenvironment-dependent, and its phenotypic plasticity in different inflammatory stages or tissue origins has not been fully elucidated (18). The clinical translation pathway for functionalization strategies is unclear, lacking a systematic evaluation from *in vitro* validation to intranasal delivery (19, 20). Data on engineered exosomes regarding nasal mucosal barrier penetration efficiency, target cell selectivity, and *in vivo* metabolic kinetics remain very limited (21, 22). These gaps urgently require an integrative approach to elucidate the mechanistic logic and complementary relationships of different modification strategies to clarify the priority directions for future translational research.

This review focuses on the application progress and mechanisms of functionalized stem cells and their derivatives in the treatment of AR, highlighting the immune regulatory mechanisms of MSCs, functional modification strategies for stem cells and their derivatives, and cutting-edge design of delivery systems and combination therapy applications. Through cross-sectional comparison and longitudinal integration of the above evidence, this article aims to provide a theoretical framework for precision cell therapy of AR and identify weak links in the current evidence chain to guide subsequent mechanism validation and clinical translation research.

## 2 Pathological basis of immune imbalance and nasal mucosal barrier damage in allergic rhinitis

### 2.1 Th2/Th17/treg immune imbalance and inflammatory microenvironment

The core immunopathological feature of AR is an imbalance in the ratio of Th2, Th17 and Treg cells. This imbalance drives the continuous disruption of the local inflammatory microenvironment in the nasal cavity and constitutes a key target for stem cell immunomodulatory therapy (23–26). Clinical sample analysis showed that patients with AR had an increased proportion of Th2 cells and a decreased proportion of Treg cells in their peripheral blood, resulting in a significant imbalance in the Th2/Treg ratio (27). Further research also found that this imbalance extended to the Th17/Treg axis. In an ovalbumin (OVA)-induced AR rat model, the proportion of Th17 cells and serum interleukin (IL)-17 levels were increased, while the proportion of Treg cells and IL-10 levels were decreased, and the Th17/Treg balance shifted toward a pro-inflammatory direction (28). This dual imbalance is not an isolated event, but rather interconnected through a common signaling pathway. Researchers used an *in vitro* co-culture system of peripheral blood mononuclear cells (PBMCs) from AR patients and found that adipose-derived mesenchymal stem cell-derived exosomes (ADSC-exos) could reverse the Th2/Treg imbalance by inhibiting the phosphoinositide 3-kinase (PI3 K)/AKT/mammalian target of rapamycin (mTOR) pathway, suggesting that PI3 K/AKT/mTOR signaling is a key node connecting metabolic state and T cell differentiation (27). Meanwhile, researchers found in an AR rat model that adipose-derived mesenchymal stem cells (ADSCs) promote Treg cell differentiation and inhibit Th17 cells by activating the IL-2/Janus kinase 3 (JAK-3)/signal transducer and activator of transcription 5 (STAT-5) signaling pathway, thereby restoring immune homeostasis (28). These two studies revealed the molecular mechanisms by which stem cells regulate the Th2/Th17/Treg balance from the PI3 K/AKT/mTOR and JAK-STAT pathways, respectively. However, the former focuses on exosome-mediated paracrine effects, while the latter emphasizes the synergistic effect of direct cell contact and signaling pathway activation.

Immunometabolic reprogramming often mediates an imbalance of Th1/Th2/Th17/Treg (23, 24). In asthma, a disease that shares immune mechanisms with AR, macrophage polarization is closely related to its metabolic patterns. The M1 type, dominated by glycolysis, often promotes neutrophil inflammation, while the M2 type, dominated by fatty acid oxidation, tends to support type 2 inflammation (25). Metabolites such as short-chain fatty acids (SCFAs) and amino acids in the nasal mucosal microenvironment can affect the Th17/Treg balance by regulating T cell mTOR and AMPK signaling (26). In addition, gut microbiota dysbiosis can alter the immune homeostasis of the distal nasal mucosa via the gut-nasal axis (GNA), while probiotics often promote Treg differentiation and inhibit Th2 responses by increasing SCFA production (27, 28). This evidence suggests that immune metabolic reprogramming (MR) or environmental factors influence immune imbalance in AR, and also provides potential targets for metabolic regulatory molecules (such as miRNAs) carried by mesenchymal stem cell-derived exosomes. Given that direct research on immune metabolism in AR is still less extensive than in asthma, most evidence comes from indirect inferences from disease models.

### 2.2 Nasal mucosal barrier damage and epithelial immune sentinel function

The structural integrity of the nasal epithelial barrier and the immune sentinel function of epithelial cells play a central role in the pathogenesis of AR (29, 30). Barrier disruption not only increases allergen permeability but also initiates and amplifies the type 2 inflammatory cascade through epithelial alarmins (31, 32). Furthermore, Treg dysfunction and epithelial barrier damage can synergistically exacerbate inflammation, and long-term chronic inflammation can also induce structural remodeling changes such as damage to the nasal mucosal epithelial barrier (33–35). Nasal mucosal epithelial cells maintain the physical barrier through tight junction (TJ) proteins (such as occludin, claudins, ZO-1) and adhesion junctions. In patients with AR, the expression of these proteins is downregulated in the nasal mucosa, the interepithelial spaces are widened, and the barrier function is impaired (29, 36). Using nasal mucosal biopsy tissue from AR patients and *in vitro* cultured human nasal epithelial cells (HNECs), it was found that MSC-derived small extracellular vesicles (MSC-sEVs) can restore epithelial barrier integrity by delivering miR-143 to target GSK3B, upregulating ZO-1 and occludin expression (36). This finding directly establishes a molecular link between stem cell paracrine products and barrier repair, and the miR-143-GSK3B axis may become a new target for AR therapy.

Epithelial cells, as immune sentinels, mainly sense stimuli and release alarm factors through pattern recognition receptors (PRRs) (37–39). When allergens break through the barrier, epithelial cells are activated and release IL-25, IL-33 and TSLP, which in turn activate type 2 innate lymphocytes (ILC2) and dendritic cells, initiating the Th2 polarization response (29, 32). Calcium signaling plays a regulatory role in this process: increased intracellular calcium concentration can activate the NF-*κ*B and AP-1 pathways to promote alarm factor transcription, while also regulating the assembly and disassembly of tight junction proteins (29). Therefore, calcium signaling disorders may have a dual effect of damaging the epithelial barrier and amplifying inflammation. Furthermore, epithelial barrier damage and immune imbalance often form a positive feedback loop: downregulation of tight junction protein expression by Th2 cytokines (IL-4, IL-13) can exacerbate barrier damage, which in turn promotes allergen penetration and persistently activates epithelial immune function (26, 40). This potential vicious cycle may be one of the mechanisms underlying persistent and difficult-to-treat AR, suggesting that stem cell therapy may need to address both barrier repair and immune regulation.

In addition to the physical barrier, nasal epithelial cells secrete mucus, antimicrobial peptides, etc., which constitute a chemical barrier (41–43). Among them, the function of hyaluronic acid (HA) depends on its molecular weight. High molecular weight HA maintains tissue homeostasis, while low molecular weight HA accumulates in inflammation and activates immune cells (44). AR patients often have abnormal HA metabolism in their nasal mucosa. The increased proportion of low molecular weight HA may activate epithelial pro-inflammatory signals through CD44 receptors; while MSCs may be able to remodel this microenvironment by secreting high molecular weight HA and regulating the expression of HA synthase (45). Currently, there is limited research on the specific mechanisms of HA in the nasal mucosa of AR, and most of the evidence comes from extrapolation from skin and airway diseases.

### 2.3 Immune metabolic reprogramming and neuroimmune cross-regulation

In addition to adaptive immunity and the epithelial barrier, the development of AR is often accompanied by neuro-immune interactions and immunometabolic reprogramming (46). Mast cells (MCs) are often distributed near blood vessels and nerve endings in the nasal mucosa. In addition to triggering immediate-phase symptoms through the classic IgE/Fc*ε*RI pathway, they can also mediate the amplification of neurogenic inflammation by responding to neuropeptides such as substance P through the MRGPRX2 receptor. This may explain the phenomenon that emotional stress exacerbates AR (47). In AR mice, the frequency of contact between MCs and nerve fibers is increased, suggesting that the formation of neuroimmune synapses may be the structural basis for maintaining chronic inflammation (48). Currently, the therapeutic potential of MRGPRX2 antagonists in AR remains to be verified.

Meanwhile, macrophage polarization is closely related to its metabolic patterns. Glycolysis-driven M1 type tends to promote Th17 responses, while oxidative phosphorylation-supported M2 type is more involved in Th2 inflammation (25). Macrophages in the nasal mucosa of those with AR often exhibit mixed polarization, accompanied by metabolic reprogramming such as enhanced glycolysis (26). As a form of innate immune memory, training immunity can alter the reactivity of monocytes/macrophages through epigenetic reprogramming. For example, the increased risk of AR in children after SARS-CoV-2 infection may be partly attributed to virus-induced training immunity increasing the sensitivity of innate immune cells to allergens (49), which also suggests that stem cell therapy may need to consider resetting epigenetic imprints.

Furthermore, neuroimmune regulation also involves interorgan communication. Skin barrier damage, Th2/Th17 imbalance, and microbial metabolites in patients with atopic dermatitis (AD) can affect the distal nasal mucosa via the circulatory system, promoting the “Atopic March” (34, 50, 51). In this process, cannabinoids exert a regulatory effect by inhibiting MC degranulation and neuropeptide release through sensory nerves and CB1/CB2 receptors expressed by MSCs (51). These findings suggest the possibility of transorganism regulation by MSCs via extravesicles. Given the current *in vivo* functional validation of neuroimmune interactions in AR, with most mechanisms still based on *in vitro* experiments or extrapolation from skin diseases, further AR-specific research is needed.

## 3 Research on stem cells in rhinitis

### 3.1 Stem cells in mucosal and airway tissues

Stem cells are defined by their capacity for self-renewal and multi-lineage differentiation and encompass embryonic stem cells, adult stem cells, and induced pluripotent stem cells (iPSCs) (52–54). Among these, mesenchymal stem cells (MSCs)—adult stromal cells present in bone marrow, adipose tissue, umbilical cord, tonsils, and nasal mucosa—are the most studied in allergic disease, because their therapeutic potential rests primarily on immunomodulation and tissue repair rather than on target-cell differentiation, with emphasis having shifted from cell replacement toward paracrine mechanisms (55, 56). The mucosal and airway stem cell populations of greatest relevance to allergic rhinitis are discussed below.

### 3.2 Establishment of the nasal cavity stem cell system (1980s–2013)

In the 1980s and 1990s, Graziadei and Monti Graziadei discovered that olfactory sensory neurons in the olfactory nerve epithelium can regenerate throughout life and are replenished by basal cells, thus providing the first evidence of the existence of an adult stem cell pool in the nasal cavity of mammals (57). In 2001, Roisen et al. first isolated olfactory nerve stem cells from adult nasal biopsy tissue (50). Between 2009 and 2011, researchers further identified olfactory ectodermal mesenchymal stem cells (OE-MSCs) in the lamina propria of the human olfactory mucosa. These cells express mesenchymal markers such as CD73 and CD90 and possess the ability to differentiate into adipogenic, osteogenic, and neurogenic cells (51). In 2013, Féron et al. published a standardized protocol for isolating adult stem cells from the human olfactory mucosa (58), marking the basic maturity of the methodology in this field. Subsequently, Duan and Lu systematically reviewed the multidirectional repair potential of four major stem cell populations in the olfactory mucosa: horizontal basal cells, globular basal cells, MSCs, and olfactory ensheathing cells (50).

### 3.3 The intersection of MSC immune regulation and allergic rhinitis research (2015–2023)

The immune-regulatory role of MSCs in AR was first mechanistically defined through suppressor of cytokine signaling 3 (SOCS3): upregulated by interferon (IFN)-*γ*, SOCS3 mediates the anti-proliferative effect of MSCs on T cells and B cells, and its blockade reverses the therapeutic effect of MSCs in an AR mouse model (59). This cell-contact-dependent effector function was later complemented by a paracrine, cell-free mechanism: MSC-derived small extracellular vesicles (MSC-sEVs) regulate dendritic cell function through the IL-10 pathway, reducing the release of Th2 cytokines (IL-4, IL-9, IL-13) in AR patients and increasing the proportion of Tregs (60). The same group subsequently extended this finding to a general immune-regulatory network of MSCs and MSC-sEVs across asthma, atopic dermatitis and AR, proposing that sEVs may replace living cells as a cell-free therapeutic direction (61). The progression from SOCS3-mediated contact inhibition to sEV-mediated paracrine regulation thus reflects a conceptual shift from cell-based to cell-free therapy; the two lines of evidence nonetheless use different readouts (T/B-cell proliferation versus DC/T-cell function) and have not been directly compared within a single model.

### 3.4 Deepening preclinical research and exploring clinical translation (2022–2025)

Clinically, this period saw a transition from sporadic animal experiments to evidence-based evaluation, marked by the registration of the first systematic review and meta-analysis protocol for MSC therapy in AR (62) and a meta-analysis of MSC efficacy across AR animal models (63). A systematic review of 74 experimental rhinitis treatments further identified stem cell therapy as one of the emerging directions (64). These syntheses, however, consistently note that the underlying studies are heterogeneous in cell source, dose, and outcome measures and are dominated by small-animal OVA models, which limits the certainty of the pooled estimates. In parallel, a proof-of-concept study transplanted iPSC-derived airway epithelium into the rat nasal cavity, providing principle validation for hereditary nasal diseases such as cystic fibrosis and primary ciliary dyskinesia (65); because AR is a non-hereditary inflammatory condition, the translational relevance of this strategy to AR remains indirect. Recent animal studies have further corroborated the efficacy of ADSCs and their exosomes in AR models (15, 66, 67), although these remain preclinical and do not yet address long-term safety or clinical dosing.

In recent years, there have been an increasing number of animal model studies evaluating the therapeutic potential of MSCs for allergic rhinitis (63). In addition to MSCs from various tissues such as bone marrow, umbilical cord, and adipose tissue, which are currently widely studied, MSCs from tonsils, human nasal mucosa, and stem cells from human exfoliated deciduous teeth (SHEDs) have also shown similar effects in regulating immunity and improving allergy symptoms (68–71). However, the efficacy and safety of MSC therapy for allergic rhinitis remain controversial and require further evaluation through systematic reviews and meta-analyses (62) (Figure 1).

**Figure 1:**
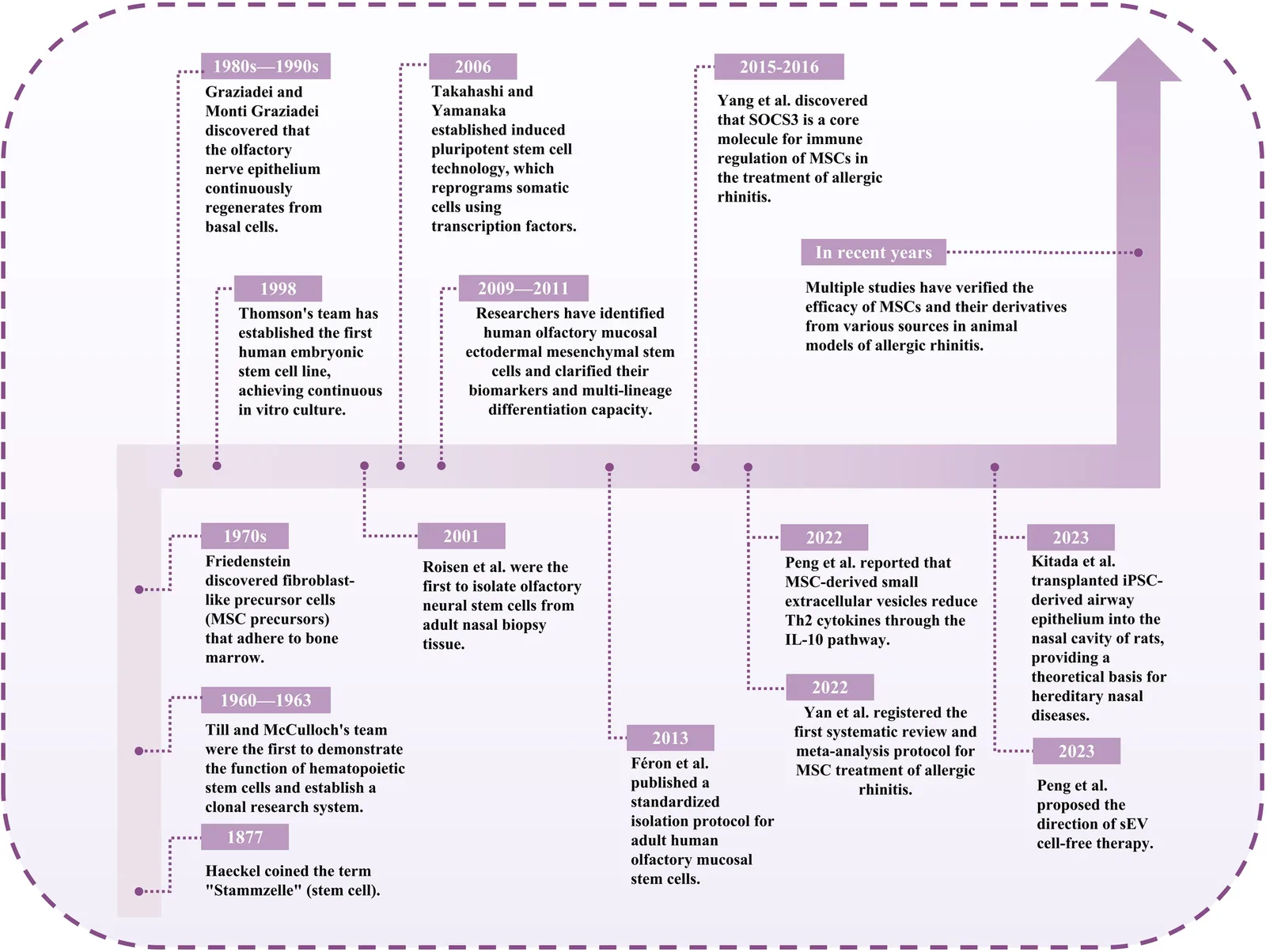
Timeline of stem cell and derivative research progress in allergic rhinitis therapy.

## 4 Stem cell functionalization therapy program

The lack of targeting and low delivery efficiency of natural MSCs are the core obstacles to their clinical application. Although MSCs can migrate to sites of inflammation and injury, the retention rate of intravenously injected MSCs in nasal mucosa in AR models is usually less than 1% of the injected dose, and most cells are captured and cleared by the pulmonary capillary bed within 24–48 h (72, 73). Furthermore, the survival rate and paracrine activity of MSCs in the *in vivo* microenvironment are affected by inflammatory states; severe inflammatory environments may induce MSC apoptosis or functional exhaustion (74). To overcome these limitations, researchers have attempted to enhance the efficacy of MSCs through functionalization (Figure 2 and Table 1).

**Figure 2:**
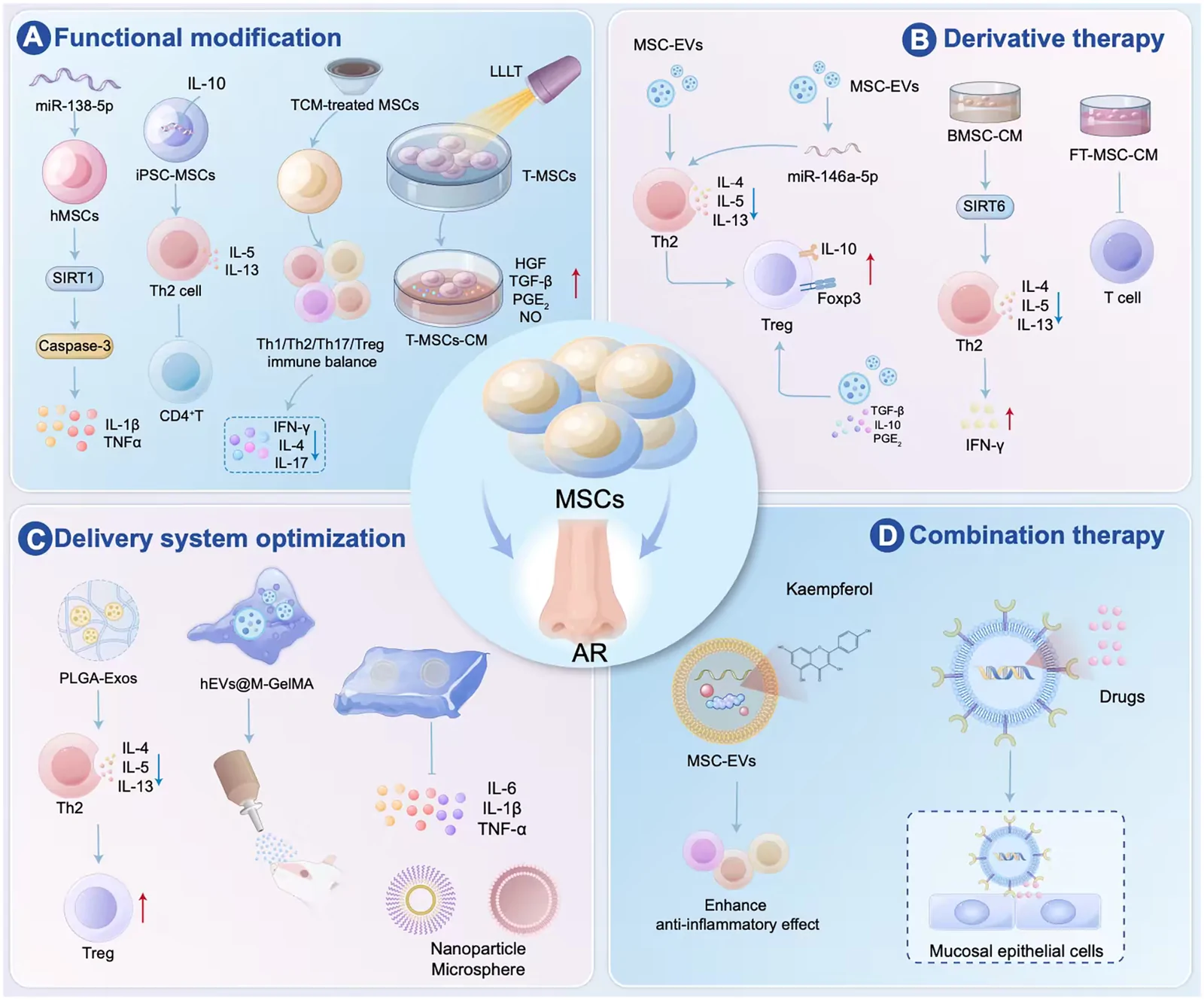
Classification and technical framework of functional modification, derivative preparation, delivery system optimization and combination therapy strategies of MSCs for allergic rhinitis treatment. Panel **(A)** shows MSC functional modification approaches including genetic engineering, drug pretreatment, and LLLT-based physical pretreatment; LLLT-treated T-MSCs produce anti-inflammatory mediators in T-MSCs-CM to restore Th1/Th2/Th17/Treg immune balance. Panel **(B)** illustrates cell-free MSC-derived therapeutics, including MSC-EVs, BMSC-CM, and FT-MSC-CM, where MSC-EVs deliver microRNAs to suppress Th2-responses and promote Treg differentiation. Panel **(C)** depicts optimized intranasal delivery carriers: PLGA-Exos, hEVs@M-GelMA, hydrogel microspheres and nanoparticles, which improve local mucosal retention and inhibit pro-inflammatory cytokines. Panel **(D)** presents combination-therapy strategies using kaempferol-loaded MSC-EVs and drug-carrying nanoparticles to achieve enhanced anti-inflammatory effects targeting mucosal epithelial cells.

**Table 1:** Comparison of major strategies for functionalized stem cells and their derivatives in AR therapy.

Strategy type	Specific methods	Mechanism	Advantages	Limitation	References
Genetic engineering modification	Control of gene expression, multi-gene co-modification	Upregulation of SIRT1 inhibits HMGB1/TLR4; inhibits Th2 response; IL-10 mediates immune regulation.	Highly targeted and capable of persistently regulating specific pathways	Long-term safety, tumorigenicity risk, and off-target effects remain unclear.	(84–86)
Drug/compound pretreatment	Pretreatment of Chinese herbal extracts, quercetin, and gallol	Regulates the balance of Th1/Th2/Th17/Treg; anti-inflammatory through the IL-10/JAK/STAT pathway.	High safety, no genetic manipulation required	Pretreatment conditions are not standardized, and active ingredients are complex.	(87, 88, 91)
Physical pretreatment	Low-Level Light Treated	Enhance the secretion of factors such as HGF, TGF-*β*, PGE₂, and NO.	Non-genetic manipulation, simple to operate	The mechanism is unclear, and there is a lot of room for parameter optimization.	(92)
Derivative therapy	MSC exosomes (containing miRNA), conditioned medium	miR-146a-5p inhibits Th2; regulates the STAT6 pathway; and promotes Treg differentiation.	Low immunogenicity	Poor targeting, rapid *in vivo* metabolism, and insufficient batch-to-batch consistency.	(68, 69, 76, 89, 90, 93)
Delivery system optimization	PLGA encapsulation, GelMA hydrogel, nanocomposite hydrogel, smart hydrogel	Prolonged local retention allows for slow or on-demand release.	Improve bioavailability and reduce dosing frequency	The biocompatibility of the material and the safety of its degradation products need to be verified.	(20, 78, 90, 94, 95)
Combination therapy	Exosomes loaded with anti-inflammatory drugs/natural products; combined with probiotics	Synergistic anti-inflammatory effects, regulating the gut-nasal axis	Multi-target synergy, potentially enhancing therapeutic efficacy	The synergistic mechanism of multiple components is complex and difficult to control.	(19, 21, 27, 82, 83)

### 4.1 Functional modification

Genetic engineering modification is one of the commonly used functional modification methods. Through this method, stem cells can be overexpressed with a beneficial gene or knocked down/suppressed with a harmful gene, thereby enhancing stem cell efficacy (71, 72). Studies have found that miR-138-5p-modified human mesenchymal stem cells (hMSCs) can alleviate nasal symptoms in mice with OVA-induced allergic rhinitis, and reduce histamine, OVA-specific immunoglobulins, and inflammatory factors in serum and nasal lavage fluid compared to unmodified hMSCs. Its effects include the ability of miR-138-5p to relieve its targeting inhibition of sirtuin 1 (SIRT1) and upregulate SIRT1, thereby inhibiting high mobility group box 1 (HMGB1)/Toll-like receptor 4 (TLR4) and its related myeloid differentiation primary response 88 (MyD88) signaling. At the same time, under the stimulation of tumor necrosis factor (TNF)-*α* and IL-6, it can reduce the downregulation of SIRT1 in hMSCs and related changes such as caspase-3, IL-1β, and TNF-α (73). However, miR-138-5p is not the only miRNA that regulates SIRT1, and miR-138-5p has other molecular targets besides SIRT1. Further research is needed to investigate other molecular targets of miR-138-5p in hMSCs besides SIRT1 in order to more comprehensively elucidate this AR treatment strategy (74–76). Th2 is often an important target in immunotherapy for allergic diseases (77). By transfecting iPSC-derived mesenchymal stem cells with lentiviral transfection to overexpress IL-10 and perform functional modification, Th2 response and the production of IL-5 and IL-13 are mainly inhibited through IL-10-mediated immunomodulation, thereby alleviating allergic airway inflammation in mice and reducing the Th2 response of AR-derived CD4⁺T cells in patients (78). Another study showed that functionalized mesenchymal stem cells can carry multiple genes and have advantages in regulating Th2 immune responses and airway inflammation, which also provides a reference for efficient treatment strategies for allergic rhinitis with similar pathogenesis (5, 79).

In the strategy of functional modification of stem cells, drug or compound pretreatment is a mild and effective means that can significantly enhance the immunomodulatory and anti-inflammatory capabilities of stem cells while avoiding the potential risks of genetic engineering (80). By using *in vitro* co-culture, specific drugs or bioactive molecules can pre-activate stem cells. This method aims to optimize the functional phenotype of cells in advance, thereby improving their subsequent therapeutic performance *in vivo*. For example, in one study, researchers prepared MSCs pretreated with extracts of the traditional Chinese medicine “Qi-fang-bi-min-tang” and applied them to an animal model of OVA-induced AR. Compared with the control group, this pretreatment strategy significantly alleviated AR symptoms, improved nasal mucosal pathological changes, effectively regulated the Th1/Th2/Th17/Treg immune balance, reduced the levels of inflammatory factors such as IFN-*γ*, IL-4, and IL-17 in plasma, and reduced the secretion of serum IgE and histamine, demonstrating a superior therapeutic effect (81). In studies of other diseases, pretreatment of stem cells with quercetin (QUR) and pyrogallol has been shown to improve acute inflammation (82, 83). These studies suggest that drug or compound pretreatment is a feasible functional modification pathway, providing important insights for future research into more traditional Chinese medicine pretreatment strategies for MSCs. Physical pretreatment is another commonly used stem cell functionalization strategy. One study showed that physical pretreatment of tonsil-derived mesenchymal stem cells (T-MSCs) with low-level light therapy (LLLT) enhanced the secretion of immunomodulatory factors hepatocyte growth factor (HGF), transforming growth factor (TGF)-β, prostaglandin E₂ (PGE₂), and nitric oxide (NO) in T-MSCs conditioned medium (T-MSCs-CM), ultimately demonstrating a stronger anti-allergic effect in a mouse model of allergic rhinitis (84). As a non-genetic intervention strategy, physical pretreatment avoids the introduction of exogenous genes while also possessing good biosafety.

### 4.2 Derivative therapy

The key to the regulation of the immune response in AR by MSCs lies in their paracrine activity, in which bioactive molecules such as miRNAs carried by exosomes play a central role. Exosomes help restore the Th2/Treg immune balance by inhibiting Th2-type inflammatory responses and promoting regulatory T cell (Treg) differentiation. Animal experiments have shown that MSC exosomes can significantly downregulate the expression of Th2-related cytokines (IL-4, IL-5, IL-13) and upregulate the levels of Treg markers (Foxp3, IL-10) in AR models, thereby reducing eosinophil infiltration in the nasal mucosa and improving symptom scores (63, 85, 86). This phenomenon has been observed in studies of MSC exosomes from different sources, including fat, bone marrow, umbilical cord, and tonsils, indicating that this regulatory mechanism is universal (63, 85, 86). At the molecular level, MSC exosomes mainly regulate the Th2/Treg differentiation signaling pathway by delivering specific miRNAs. For example, human bone marrow MSC exosomes can target the SERPINB2 pathway by carrying miR-146a-5p, thereby inhibiting Th2 cell differentiation (87). In a co-culture system of peripheral blood CD4+ T cells from AR patients, exosome treatment significantly reduced the proportion of Th2 cells and increased the proportion of Treg cells (87), a finding consistent with subsequent research results on ADSC exosomes (88). In addition to miRNAs, cytokines such as TGF-*β*, IL-10 and PGE2 secreted by MSCs also participate in the induction and amplification of Tregs, forming a dual regulatory network together with exosomes (63). Although the dosage, frequency and evaluation time points of different studies may vary, which may lead to fluctuations in the intensity of the effect, the overall trend of exosome-induced Th2/Treg balance shifting towards Treg remains consistent. Notably, the signaling mediators are not consistent across MSC sources: ADSC exosomes were reported to improve the Th2/Treg ratio through the PI3 K/AKT/mTOR axis (66), whereas PI3K-AKT pathway activation was found to weaken immunomodulatory efficacy in a tonsil MSC senescence model (75), suggesting that these pathway effects are source- and context-dependent.

Current research limitations in this field primarily lie in the fact that most studies focus on changes in the proportion of immune cell subsets after exosome treatment, with insufficient validation of direct binding between exosomal miRNAs and target genes. Secondly, the synergistic or antagonistic effects between exosomes and other paracrine factors in the *in vivo* microenvironment have not been systematically elucidated. The precise molecular network regulating Th2/Treg balance by MSC exosomes still awaits confirmation through further functional perturbation experiments.

Conditioned culture media of natural MSCs can exert anti-inflammatory effects through immunomodulation in animal models of AR. In AR models, conditioned medium from bone marrow-derived mesenchymal stem cells (BMSCs), termed BMSC-CM can regulate immune responses through signal transduction and the STAT6 signaling pathway, significantly reducing OVA-induced eosinophil infiltration and Th2 cytokine (IL-4, IL-5, IL-13) levels in the nasal mucosa of AR mice, while upregulating IFN-*γ* expression, suggesting that it exerts its anti-inflammatory effect by correcting the Th1/Th2 imbalance (76). In another study, tonsil-derived mesenchymal stem cell conditioned medium in a mouse model of allergic rhinitis partially alleviated allergy symptoms and nasal mucosal inflammation by inhibiting T cell-related activation (69). Conditioned culture media containing MSCs, through immunomodulatory anti-inflammatory effects, hold promise as a safe and cell-free novel therapeutic strategy for allergic rhinitis. Currently, research on functionalized MSC exosomes in AR treatment remains primarily at the proof-of-concept level, with insufficient data on large-scale production processes, batch consistency, and long-term safety. This strategy partially improves the efficacy bottleneck of natural MSCs by enhancing MSC survival and anti-inflammatory factor secretion, but the long-term safety and potential tumorigenic risks of gene modification still require systematic evaluation (77). Furthermore, most *in situ* gel research remains at the small animal model stage, and the feasibility of its translation to large animals and clinical applications, the design of drug delivery devices, and the large-scale production process have not yet been fully validated. (Table 2)

**Table 2:** Comparison of mechanisms and effects of MSCs and their derivatives from different sources in AR research.

MSC source	Research Model	Main mechanism of action	Key effector molecules	Effect	References
ADSC	OVA-induced AR rats; co-culture of PBMCs from AR patients	Activation of the IL-2/JAK-3/STAT-5 pathway promotes Treg differentiation; exosomes improve the Th2/Treg ratio via PI3 K/AKT.	IL-2、JAK-3、STAT-5；PI3 K/AKT	Restoring the Th17/Treg balance and reducing inflammation	(66, 67)
BMSC	AR mouse model; CD4⁺ T cells in peripheral blood of AR patients	Conditioned culture media regulate Th1/Th2 via the STAT6 pathway; exosomes inhibit Th2 differentiation and promote Treg differentiation.	STAT6; exosomes (containing miRNA)	Reduce eosinophil infiltration and correct Th1/Th2 imbalance	(76, 96)
hUCMSC	Mouse colitis (AR extrapolation), acute lung injury (extrapolation)	Anti-inflammatory effects are achieved through the IL-10/JAK/STAT signaling pathway; gallol enhances anti-inflammatory efficacy.	IL-10、JAK、STAT	Modulate and remodel inflammatory cytokines to reduce inflammation	(88, 91)
T-MSC	AR mouse model	Inhibits T cell activation; LLLT enhances the secretion of HGF, TGF-β, PGE₂, etc.	HGF、TGF-β、PGE₂、NO	Relieve allergy symptoms and nasal mucosal inflammation	(69, 92)
SHED	Mouse AR model; *in vitro* co-culture of PBMCs from AR patients	TGF-β1 promotes Foxp3⁺Treg expansion, inhibits T cell proliferation and Th2/Th17 inflammation, upregulates IFN-*γ*, and restores CD4⁺T immune balance.	TGF-β1、IL-10、PGE2、Foxp3	Reduces serum OVA-specific IgE and IgG₁; inhibits Th2-type inflammatory response; induces Treg amplification *in vitro*.	(71)

ADSC, adipose-derived mesenchymal stem cell; BMSC, bone marrow-derived mesenchymal stem cell; hUCMSC, human umbilical cord mesenchymal stem cell; T-MSC, tonsil-derived mesenchymal stem cell; SHED, stem cell from human exfoliated deciduous teeth.

### 4.3 Delivery system optimization

To overcome the limitations of natural MSC exosomes, such as poor *in vivo* stability, low targeting efficiency, and short local retention time, the development of novel delivery systems has become an important direction for enhancing the efficacy of AR immunotherapy. In AR treatment, poly(lactic-co-glycolic acid) (PLGA)-encapsulated MSC exosomes (PLGA-Exos) have been shown to prolong the local retention time of exosomes in the nasal cavity through a sustained-release effect, thereby enhancing the immunosuppressive effect (86). This study showed that after nasal administration of PLGA-Exos, the levels of Th2 cytokines (IL-4, IL-5, IL-13) in AR mice were significantly reduced, and the proportion of Tregs was increased, with better results than the free exosome group.

Hydrogel delivery systems provide another effective platform for the localized and sustained release of MSC exosomes. MXene-modified gelatin methacryloyl (GelMA) hydrogel loaded with hypoxia-induced MSC exosomes (hEVs@M-GelMA) exhibited excellent biocompatibility and sustained-release performance in an AR mouse model (89). The porous structure of this hydrogel facilitates the uniform distribution and sustained release of exosomes, allowing a single nasal administration to maintain an immunomodulatory effect for at least 7 days, significantly reducing the frequency of administration. Furthermore, the nanocomposite hydrogel system combines the natural anti-inflammatory components of arnica extract and propolis nanoparticles with menstrual blood-derived MSCs, demonstrating a synergistic inhibitory effect on IL-6, IL-1β, and TNF-α in *in vitro* experiments (90). This multi-component delivery system, by integrating anti-inflammatory active substances from different sources, may achieve a more comprehensive immune-regulating effect.

Another advantage of the novel delivery system is that it can enhance the targeting and penetration capabilities of exosomes. Nanoparticles, as auxiliary delivery carriers for exosomes, can improve the retention and absorption efficiency of exosomes in the nasal mucosa (78). Hydrogel microspheres (HMs) have attracted attention as cell and drug delivery platforms for the application of AR therapy. Their injectability and biodegradability provide flexible design space for local immune regulation (79). In AR animal models, intelligent delivery systems have shown initial potential for delivering stem cells or exosomes. Thermosensitive hydrogels (such as poloxamer 407/188) rapidly gel at nasal temperatures, allowing for localized sustained release of encapsulated MSCs or exosomes, maintaining effective concentrations within the nasal mucosa. Compared to free forms, gel delivery reduces dosing frequency and protects the load from enzymatic degradation. pH-responsive gels can trigger release at low pH levels in the inflammatory microenvironment, enabling on-demand drug delivery (20).

However, most current *in-situ* gel studies are limited to small animal models. When it comes to promoting them to large animals and clinical applications, the design of the drug delivery device and the large-scale production process still require further validation. The biocompatibility and degradation rate of the gel material should be matched with the therapeutic window. Excessive cross-linking may hinder the release of cells or exosomes, and degradation products may also induce local inflammation (20). Delivery systems are also mostly in the preclinical exploration stage, and there is a lack of systematic cross-sectional comparisons of the regulatory effects of different modification strategies on immune cell subsets; in terms of biosafety, the long-term impact assessment of immunogenicity and degradation products is also insufficient. In addition, the dosage conversion and dosing regimens from animal experiments to clinical applications still need to be gradually optimized based on actual conditions.

### 4.4 Combination therapy

Unlike drug-pretreated stem cells, MSCs and their exosomes can be designed as delivery platforms to carry anti-inflammatory drugs (such as glucocorticoids and plant active ingredients) and immunomodulatory molecules to achieve synergistic therapy (19, 21). For example, loading natural products with anti-inflammatory activity, such as kaempferol and chlorogenic acid compounds, into MSC exosomes can improve the bioavailability of drugs in the nasal mucosa by utilizing the biocompatibility and transmucosal barrier capacity of exosomes, while enhancing the anti-inflammatory effect through the inherent immunomodulatory effects of MSC derivatives (80, 81). Nanoparticle-mediated targeted delivery systems, by encapsulating anti-inflammatory drugs or gene fragments in biodegradable nanocarriers and coupling them with nasal mucosal epithelial cells or immune cell-specific ligands, can achieve drug enrichment and controlled release at the lesion site (21). Drug-loaded combination therapy strategies can compensate for the insufficient targeting of natural MSCs and their derivatives in AR treatment, providing a feasible direction for next-generation stem cell therapy.

Furthermore, flavonoids (such as quercetin and luteolin) have been found to exert immunomodulatory effects by regulating training-related signaling pathways (82), providing a theoretical basis for encapsulating natural compounds and stem cell derivatives in intranasal gels or microneedles. Multi-omics fusion reveals the role of the “gut-nasal axis” in AR, suggesting that gut microbiota metabolites may influence MSC efficacy through training immunity, implying the potential for future probiotic interventions (27, 83).

## 5 Future research directions

The core of the immunopathology of AR lies in the vicious cycle of Th2/Th17/Treg cell imbalance and nasal mucosal barrier damage, while functionalized stem cells and their derivatives show therapeutic potential by targeting and regulating this immune mechanism. The existing evidence can be summarized as follows: (1) The proportion of Th2 and Th17 cells and the proportion of Treg cells are increased in the peripheral blood and local tissues of AR patients. The imbalance of Th2/Treg ratio is positively correlated with the severity of the disease (66, 97); (2) MSCs mainly inhibit Th2-type cytokines (IL-4, IL-13) and promote Treg differentiation through paracrine pathways, especially through the miRNAs carried by exosomes (such as miR-146a, miR-21, etc.), thereby restoring the Th2/Treg balance (18, 66); (3) Although natural MSCs can reduce inflammation in AR animal models, there are bottlenecks such as insufficient targeting, low delivery efficiency and limited cell survival (75); (4) Novel delivery systems have further solved the problem of short local retention time of exosomes in the nasal cavity and enhanced bioavailability (22).

Current research has many key limitations and controversies. First, the vast majority of evidence comes from mouse models (induced by OVA or house dust mites). Animals and humans have significant differences in nasal anatomy, immune cell subsets, and microbiome composition, making it difficult to directly extrapolate animal efficacy to clinical practice (18, 98). Secondly, existing clinical studies generally have small sample sizes, mostly 10–30 cases, lack multicenter, randomized, double-blind designs, and the follow-up period is usually no more than 3 months, making it impossible to assess long-term safety (27, 99). Third, the heterogeneity of MSC sources (bone marrow, adipose tissue, umbilical cord, tonsils, etc.) and culture conditions (such as passage number, oxygen concentration, and culture medium composition) makes direct comparison of results between studies difficult. For example, Han et al. observed that ADSC exosomes may improve the Th2/Treg ratio through PI3 K/AKT/mTOR (27), while Qiu's team found that PI3K-AKT pathway activation may weaken immunomodulatory efficacy in a tonsil MSC aging model (99). Furthermore, a universally accepted regulatory framework remains lacking for the standardized production and quality control of engineered exosomes (19). Another point of contention is whether the immune regulation of MSCs is primarily “activating” or “inhibiting” (100). In the subgroup of severe AR with Th17 dominance, questions such as whether simply enhancing Tregs is sufficient to reverse the pathology remain to be systematically answered.

These mechanistic conclusions, however, must be interpreted cautiously, as most supporting evidence derives from acute OVA- or house-dust-mite-induced rodent models that diverge from human pediatric AR in several respects (18, 97). First, these models rely on artificial sensitization protocols—typically intraperitoneal priming followed by high-dose nasal challenge—and therefore recapitulate neither the natural, low-dose, multi-allergen sensitization nor the chronic, relapsing course of childhood AR. Second, rodent nasal anatomy differs markedly from that of humans in turbinate structure, mucosal surface area, mucus composition, and nasal breathing physiology, all of which affect intranasal delivery, local retention, and engraftment of MSCs or their derivatives. Third, acute models seldom reproduce the chronic tissue remodeling—goblet-cell hyperplasia, subepithelial fibrosis, and basement-membrane thickening—that characterizes established human AR. These discrepancies help explain why the robust preclinical efficacy of MSCs has only rarely translated into pediatric clinical practice: children differ from adult rodents in immune maturation, in the frequent coexistence of AR with asthma and atopic dermatitis, and in the safety and ethical constraints that limit repeated intranasal administration of live-cell or engineered products. Preclinical findings should therefore be regarded as proof of mechanism rather than proof of efficacy, and validation in large-animal and chronic, age-relevant disease models is warranted before pediatric trials are undertaken.

Looking ahead to the next 3–5 years, this field will see the following breakthrough trends. First, the application of single-cell RNA sequencing and spatial transcriptomics will map the cellular atlas of the nasal mucosal immune microenvironment in AR patients, precisely locating the target cell subpopulations for MSC exosomes, thereby guiding personalized engineering design. Second, artificial intelligence and computational medicine will accelerate the rational design of exosome drug delivery systems—using deep learning to predict miRNA-target gene interaction networks, screening for optimal combinations of immunomodulatory molecules, and simulating the diffusion dynamics of exosomes in the nasal mucus layer. Third, synthetic biology tools, such as optogenetically regulated MSCs and programmable exosomes, will develop new intelligent delivery systems to achieve on-demand release of immunomodulatory factors, for example, activating anti-inflammatory programs during peak inflammation and automatically shutting them off during remission to avoid excessive immunosuppression. Fourth, the integration of multiple omics, including metabolomics, epigenomics, and microbiome, will reveal the role of the “gut-nasal axis” in AR. Gut microbiota metabolites may affect the efficacy of MSCs by training the immune system, suggesting that future therapies may require combined probiotic intervention (27, 83). Fifth, liquid biopsy, through circulating exosomal miRNA profiling, is expected to become a non-invasive tool for monitoring the efficacy of AR, and to assess the targeting efficiency and immunomodulatory effects of engineered exosomes *in vivo* in real time.

From a clinical translational perspective, functionalized stem cells and their derivatives hold promise for filling the gaps in existing AR treatments, offering a non-hormonal, non-surgical “immune reset” option for patients with moderate to severe, refractory AR, reducing the burden of long-term medication. However, caution is warranted due to the lack of evidence-based support for individualized dosage and treatment duration. For patients, engineered exosome formulations in nasal spray form will significantly improve medication adherence and avoid the tumorigenic risks associated with live cell therapy.

## 6 Pediatric perspective

Although AR is most prevalent in children and adolescents, dedicated pediatric evidence for stem cell- and EV-based strategies remains limited, constituting an important gap in the field. Pediatric AR is not simply a scaled-down version of adult disease: it develops against a background of ongoing immune maturation and a rapidly evolving microbiota, which together shape both susceptibility and treatment response. A prospective birth cohort of 411 high-risk children followed to 6 years of age showed that reduced intestinal microbiota diversity at 1 and 12 months of life was associated with an increased risk of allergic rhinitis (*P* = 0.007), allergic sensitization, and peripheral blood eosinophilia, whereas no such association was observed for asthma or atopic dermatitis (101). This disease-specific pattern indicates that early-life microbial composition is a developmental determinant of pediatric AR, and it suggests that microbiome-modifying and stem cell-based interventions in this age group should account for the timing and trajectory of immune maturation. Because sputum induction and bronchoalveolar lavage are difficult to perform in young children, EV analysis in readily accessible biofluids is especially well suited to pediatric research. In children, urine-derived bacterial EVs differ among allergic rhinitis, chronic rhinitis, atopic asthma, and healthy controls, and their profiles can distinguish these disease states (98). Further characterization of bacterial microbiota-derived urinary EVs in children with allergic airway disease revealed compositional and functional features that correlated with total IgE and blood eosinophil percentage, suggesting that these vesicles carry disease-relevant, non-invasively accessible signatures (99). Longitudinal urinary EV profiling in children with asthma likewise showed persistently altered microbial EV patterns, supporting urine as a practical surrogate for airway microbiota status in this population (100). Most directly relevant to AR, a pediatric study identified serum exosome-derived microRNAs as predictors of subcutaneous immunotherapy response: among 812 detected miRNAs, hsa-miR-4669 was significantly lower in responders and exhibited an area under the curve of 0.785 for predicting efficacy, with levels correlated with symptom severity; notably, the target genes of the differentially expressed miRNAs were enriched in pathways regulating stem cell pluripotency and Wnt signaling (102). Conducted in an otolaryngology setting, this work provides proof of principle that exosome-based liquid biopsy is feasible and informative in children with AR, and it links circulating EV cargo to the stem cell regulatory networks that are the focus of this review. Collectively, these studies establish that EVs, whether microbiota- or host-derived, can be sampled non-invasively and carry disease-relevant information in pediatric allergic airway disease; however, none has yet evaluated the therapeutic efficacy or safety of functionalized stem cells or their derivatives specifically in children. Given the distinct immune maturation, frequent comorbidity with asthma and atopic dermatitis, and more stringent safety and ethical constraints in this population, age-specific preclinical models and dedicated pediatric trials are warranted before functionalized stem cell derivatives can be translated into pediatric AR care.

## 7 Conclusion

The core pathological link in AR is a vicious cycle consisting of Th2/Th17/Treg immune imbalance and damage to the nasal mucosal barrier. MSCs and their exosomes can regulate multiple signaling pathways by delivering miRNAs, inhibiting Th2-type inflammation, promoting Treg differentiation, and repairing epithelial tight junctions. However, the poor targeting and short local retention time of natural MSCs limit their therapeutic efficacy. But through functional modification, engineered exosomes, and hydrogel delivery systems, their anti-inflammatory efficacy and bioavailability can be significantly enhanced, demonstrating therapeutic potential superior to natural cells.

Current evidence largely comes from small animal models, and clinical studies lack large-sample, long-term follow-up data. Furthermore, the heterogeneity of MSCs from different sources and under different culture conditions leads to insufficient comparability of results, and a unified framework for the standardization and safety assessment of engineered products is still lacking. Therefore, functionalized stem cell derivatives offer a promising immunomodulatory strategy for AR, but key issues such as delivery efficiency, long-term safety, and batch consistency still need to be systematically addressed before they can be successfully applied to clinical practice.
